# Estimating the birth prevalence and pregnancy outcomes of congenital malformations worldwide

**DOI:** 10.1007/s12687-018-0384-2

**Published:** 2018-09-14

**Authors:** Sowmiya Moorthie, Hannah Blencowe, Matthew W. Darlison, Joy Lawn, Joan K. Morris, Bernadette Modell, Congenital Disorders Expert Group, A. H. Bittles, H. Blencowe, A. Christianson, S. Cousens, M. W. Darlison, S. Gibbons, H. Hamamy, B. Khoshnood, C. P. Howson, J. Lawn, P. Mastroiacovo, B. Modell, S. Moorthie, J. K. Morris, P. A. Mossey, A. J. Neville, M. Petrou, S. Povey, J. Rankin, L. Schuler-Faccini, C. Wren, K. A. Yunnis

**Affiliations:** 1 PHG Foundation, 2 Worts Causeway, Cambridge, UK; 2 Centre for Maternal, Adolescent, Reproductive, and Child Health, London School of Hygiene and Tropical Medicine, London, UK; 3 Centre for Health Informatics and Multiprofessional Education (CHIME), University College London, London, UK; 4 Centre for Environmental and Preventive Medicine, Wolfson Institute of Preventive Medicine, Barts and the London School of Medicine and Dentistry, Queen Mary University of London, London, UK

**Keywords:** Congenital malformations, Prevalence, Pregnancy outcomes, Estimation

## Abstract

Congenital anomaly registries have two main surveillance aims: firstly to define baseline epidemiology of important congenital anomalies to facilitate programme, policy and resource planning, and secondly to identify clusters of cases and any other epidemiological changes that could give early warning of environmental or infectious hazards. However, setting up a sustainable registry and surveillance system is resource-intensive requiring national infrastructure for recording all cases and diagnostic facilities to identify those malformations that that are not externally visible. Consequently, not all countries have yet established robust surveillance systems. For these countries, methods are needed to generate estimates of prevalence of these disorders which can act as a starting point for assessing disease burden and service implications. Here, we describe how registry data from high-income settings can be used for generating reference rates that can be used as provisional estimates for countries with little or no observational data on non-syndromic congenital malformations.

**Electronic supplementary material:**

The online version of this article (10.1007/s12687-018-0384-2) contains supplementary material, which is available to authorized users.

## Introduction

The Modell Database of Congenital Disorders (MGDb) uses a set of defined methods to relate demographic data to known birth prevalence of selected groups of congenital disorders, in order to generate estimates relevant to public health, policy-making and clinical practice (Modell et al. 2016; Moorthie et al. 2017). We use the term baseline birth prevalence to refer to the prevalence of the disorder among all births (i.e. livebirths plus stillbirths) that would occur in the absence of any intervention. This provides the starting point for modelling the current and future prevalence of these disorders as it provides an “envelope” into which all outcomes must fit (Moorthie et al. 2017).

The existence of long-standing congenital anomaly registries has the advantage that high-quality population-based data on birth prevalence and pregnancy outcomes are readily available in settings with rigorous surveillance programmes, which provide information on congenital malformations. However, not all countries have yet established robust surveillance systems and, for these countries, methods are needed to generate estimates of prevalence of these disorders which can act as a starting point for assessing disease burden and service implication (Blencowe et al. 2017; Moorthie et al. 2017). In this paper, which is the 5th in a supplement on the estimation of congenital disorders, we describe how data from the European Surveillance of Congenital Anomalies and Twins (EUROCAT) network can be used both to obtain country-specific rates for birth prevalences and outcomes and to generate average European rates for use as reference rates for countries with little or no observational data on non-syndromic congenital malformations.

Congenital malformations are structural abnormalities of prenatal origin and can be classified into three groups based on their cause: genetic, environmental or multifactorial (Czeizel 2005). The precise cause of many malformations that fall under the multifactorial group is unknown; however, they are thought to be due to interactions between genetic and environmental factors (Czeizel 2005; EUROCAT 2004; Kurnit et al. 1987). In MGDb, congenital malformations are bundled as far as possible according to underlying cause. Malformations caused by known environmental exposures (e.g. teratogens including maternal infections) are treated separately. Malformations associated with chromosomal disorders are treated as part of the relevant chromosomal syndrome, and malformations associated with single gene disorders, genetic syndromes or parental consanguinity are grouped as inherited disorders. These steps leave a large group of congenital malformations with multifactorial or unknown cause. In MGDb, this group is modelled under the heading of non-syndromic congenital malformations (NSCM), as they are not associated with any known primary environmental or genetic syndrome. Malformations that affect only one system are called isolated malformations; malformations involving more than one system are called multiple malformations.

As our primary data source is EUROCAT, we have followed EUROCAT classification in our methodology. Thus, isolated malformations are classified as those that include more than one malformation from the same body system and sequences, included in this are single malformations (e.g. cleft lip), more than one malformations from the same system (e.g. VSD and coarctation and more than one malformation as part of a sequence (e.g. spina bifida with talipes) (EUROCAT 2013). Malformations that are not classified as above and involve more than one system fall under the multiple malformation group.

## Description of data source

EUROCAT is our principal source for the baseline epidemiology of congenital malformations because it collects data from numerous registries in European countries with advanced diagnostic facilities. The network includes registries that submit raw data which is analysed centrally and associate registries that submit aggregated data. Special reports, details of methods used and lists of publications are available via the EUROCAT website. In brief, data are collected on all major structural congenital anomalies, chromosomal anomalies, syndromes and other hereditary conditions that are associated with structural anomalies. Registration covers affected live births, foetal deaths after 20-week gestation and termination of pregnancy for foetal anomaly following prenatal diagnosis. There is variation between registries on the age up to which new cases are ascertained; however, the majority of registries ascertain cases up to at least 1 year of life (Boyd et al. 2011).

EUROCAT publishes detailed downloadable data on the web (EUROCAT). It is possible to request specific data; however, we have restricted ourselves to using the publicly available downloadable data. This is in order to develop a methodology that can be easily replicated by public health and policy-makers, utilising readily accessible data sources.

This data set was chosen as it contains standardised, comprehensive information from a range of countries on all major congenital malformations. The countries providing data have advanced facilities for diagnosis and data collection, including multiple sources (often linked) such as specialist databases for individual malformations, hospital admissions and discharge records, plus foetal neonatal and child pathology reports allowing for good ascertainment. EUROCAT has developed a number of strategies to ensure data quality, including the development of data quality indicators (DQIs) which can be compared with EUROCAT average rates to evaluate factors such as ascertainment, accuracy of diagnosis, completeness of information, availability of denominator information and timeliness of data transmission (Loane et al. 2011). Our evaluation of the data from EUROCAT shows that although there is variation in the reported prevalence rate between registries, rates from each registry are consistent over time, which may reflect differences in the pattern of data collection. Some inter-country variation is due to real differences in prevalence, for example, the higher prevalence of neural tube defects in Northern and Eastern Europe and orofacial clefts in Scandinavian populations that has been previously reported (Khoshnood et al. 2015; Mossey and Modell 2012).

## Data

Only data from the registries that are full EUROCAT members and therefore submit raw, un-aggregated data were included in the analysis. Some registries in countries where termination of pregnancy (TOPFA) is legal do not report terminations, and this would lead to under-ascertainment of total birth prevalence. We therefore exclude data from these registries. In addition, when estimating average rates for termination of pregnancy, we excluded data from Ireland and Malta where TOPFA is illegal.

EUROCAT prevalence tables available online provide information on the number of cases of all congenital malformations either including or excluding cases with a known genetic condition, and the population denominator by year; the numbers of affected births and birth outcomes (live birth, foetal death, termination for foetal anomaly). These data are further broken down to provide the number of cases under 11 system groups (e.g. nervous system, eye, congenital heart defects etc.) and diagnoses under these system groups (e.g. neural tube defect, congenital glaucoma, severe CHD); diagnoses relating to certain environmental causes (e.g. foetal alcohol syndrome, maternal infections resulting in malformations) and the number of cases of chromosomal disorders, which is further broken down by specific diagnosis (Down syndrome, Patau etc.). Each case appears only once in each system group, but cases with malformations affecting more than one system group appear in more than one system sub-group.

Data for the years 1980–2012, by country, for all congenital malformations excluding cases with known genetic conditions were downloaded (“non-genetic” cases) (EUROCAT). Birth prevalence and birth outcome rates were calculated as the number of cases per 1000 total births, with the denominator being the total births covered by the registry. Foetal deaths, defined as losses after 20 weeks of pregnancy, are used cautiously as a proxy for stillbirths in MGDb. In estimating birth outcome rates in MGDb, it is important to estimate the foetal death rate in the absence of any intervention as this provides the baseline situation allowing assessment of the impact of interventions, and also enables estimation for countries with limited or no access to prenatal diagnosis. TOPFA is common in most countries participating in EUROCAT, and hence the foetal death rate was calculated as foetal deaths per 1000 “continuing pregnancies” (i.e. per 1000 pregnancies that have not been terminated). Average European rates were derived from an unweighted average of country-specific rates.

## Calculating average global reference rates for non-syndromic congenital malformations

- Step 1: Calculating rates of non-syndromic congenital malformations (NSCM)

**Fig. 1:**
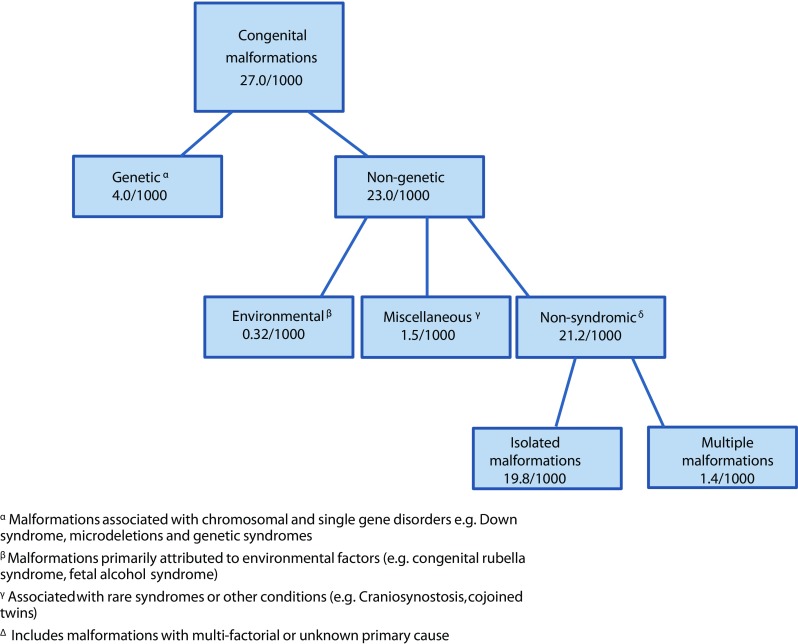
Calculating rates for non-syndromic congenital malformations from EUROCAT data. Main groups of congenital malformations included in congenital anomaly registries participating in EUROCAT (average total prevalence rates/1000, 2000–2009) are shown below. Rates for non-syndromic congenital malformations were calculated by subtracting cases attributable to environmental factors and associated with other rare syndromes from total non-genetic cases> non-syndromic congenital malformation

Data for the “non-genetic” group provide the total sum of individuals with congenital malformations including congenital malformations associated with other syndromes/anomalies, or with environmental causes. The total numbers of individuals with NSCM were obtained by subtracting cases that were present in the “other anomalies/syndromes subgroup” from the EUROCAT total for individuals with “non-genetic” malformations. This consisted of cases present in the following sub-groups: teratogenic syndromes with malformations, skeletal dysplasias, situs inversus, craniosyntosis, congenital constriction bands/amniotic band, conjoined twins, congenital skin disorders, VATER/VACTERL, vascular disruption anomalies and lateral anomalies. We have assumed that each case represents one individual for these sub-groups (Fig. 1). Details of the rates in these groups can be found in Web appendix Table 1.

The data were aggregated into 5-year groups in order to relate it to World Population Prospects (WPP) (United Nations Population Division) demographic data, and the evolution of rates for each system sub-group and totals were examined. This led to the selection of the 2000–2009 interval for calculation of the average European baseline birth prevalence, because it provides the most stable data in relation to birth prevalence. The spread of routine foetal anomaly scanning led to a gradual rise in the reporting of birth prevalences of some congenital disorders from 1980 to around 2000 (Fig. 1, Web appendix). Data from the most recent 5-year period are not included as they were incomplete (at the time the data was downloaded, 2012 was the latest year reported). The EUROCAT average for TOPFA is based on rates over the time period 2000–2009 by 5-year intervals.

- Step 2: Country-specific rates for non-syndromic congenital malformations

The above process was applied to country-specific data to obtain country-specific rates for NSCM. Table 1 shows the total prevalence rates for NSCM in 2000–2009, with countries ranked in descending order of total reported birth prevalence. Although there are substantial inter-country differences, individual country rates have remained relatively stable over time.

**Table 1:** Rates for non-syndromic congenital malformations for time period 2000–2009 by country, with birth outcomes. Total birth covered *n* = 9, 664, 000

Country	Births 2000–2009	Total NSCM /1000 [95% CI]	Outcomes /1000 [95% CI]			Outcomes %			Foetal deaths % of continuing pregnancies
			Live births	Fetal deaths	TOPFA	% Live births	% Foetal deaths	% TOPFA	
Germany	207,250	31.4 [30.65, 32.15]	29.0 [28.28,29.72]	0.43 [0.34,0.52]	2.0 [1.81,2.19]	92.4	1.4	6.3	1.46
Norway	590,723	31.2 [30.76, 31.64]	29.2 [28.77,29.63]	0.21 [0.17,0.25]	1.80 [1.69,1.91]	93.4	0.7	5.9	0.73
Switzerland	73,522	30.8 [29.54, 32.06]	27.9 [26.70,29.10]	0.30 [0.17,0.43]	2.70 [2.32,3.08]	90.4	1.0	8.6	1.06
Austria	103,719	29.5 [28.47, 30.53]	28.0 [26.99,29.01]	0.29 [0.18,0.40]	1.00[0.80,1.20]	94.5	1.0	4.5	1.03
Hungary	976,884	26.7 [26.38,27.02]	25.2 [24.89,25.51]	0.09 [0.07,0.11]	1.40 [1.33,1.47]	94.4	0.3	5.2	0.37
Malta	40,280	26.7 [25.11,28.29]	26.2 [24.63,27.77]	0.57 [0.32,0.82]	0.00[−0.01,0.01]	97.9	2.1	0	2.14
Denmark	53,491	24.2 [22.89,25.51]	21.8 [20.55,23.05]	0.43 [0.24,0.62]	2.00 [1.61,2.39]	90	1.8	8.2	1.93
Ukraine	146,055	22.0 [21.24,22.76]	18.3 [17.61,18.99]	0.80 [0.65,0.95]	2.90 [2.62,3.18]	83.3	3.6	13.1	2.81
Netherlands	191,002	21.0 [20.35,21.65]	20.0 [19.37,20.63]	0.35 [0.26,0.44]	1.00 [0.86,1.14]	94.6	1.7	3.7	1.74
Belgium	315,051	20.6 [20.10,21.10]	18.6 [18.13,19.07]	0.31 [0.25,0.37]	1.70 [1.55,1.85]	90.4	1.5	8.1	1.64
France	1,290,859	20.4 [20.16,20.64]	17.1 [16.88,17.32]	0.26 [0.23,0.29]	3.10 [3.00,3.20]	83.7	1.3	15.1	1.51
United Kingdom	2,235,229	19.5 [19.32,19.68]	16.5 [16.33,16.67]	0.48 [0.45,0.51]	2.60 [2.53,2.67]	84.4	2.4	13.1	4.19
Ireland	398,628	17.0 [16.60,17.40]	16.4 [16.00,16.80]	0.58 [0.50,0.66]	0.00 [0.00,0.00]	96.5	3.4	0	3.43
Croatia	65,263	15.7 [14.74,16.66]	14.7 [13.77,15.63]	0.17 [0.06,0.28]	0.80 [0.58,1.02]	93.7	1.1	5.3	1.13
Italy	1,237,324	15.0 [14.79,15.21]	13.2 [13.00,13.40]	0.07 [0.05,0.09]	1.70 [1.63,1.77]	88.1	0.4	11.5	0.5
Spain	1,550,570	11.5 [11.33,11.67]	10.6 [10.44,10.76]	0.11 [0.09,0.13]	0.80 [0.76,0.84]	92.2	1.0	6.9	1.04
Portugal	188,476	9.3 [8.86,9.74]	8.4 [7.99,8.81]	0.15 [0.09,0.21]	0.70 [0.58,0.82]	90.8	1.7	7.5	1.79
EUROCAT average		19.48 [19.40,19.57]	17.4 [17.32,17.49]	0.27 [0.26,0.28]	1.84 [1.81,1.86]	89.3	1.4	9.4	1.53
EUROCAT average (TOPFA legal and reported)		19.56 [19.47,19.65]	17.41 [17.33,17.49]	0.26 [0.25,0.27]	1.92 [1.90,1.95]	88.9	1.3	9.8	1.45

*NSCM*: Nonnon-syndromic congenital malformation,

*TOPFA*: termination of pregnancy for foetal anomaly,

*CI*: confidence interval

Higher than average rates are reported from a number of countries, most of them in Northern Europe. A higher prevalence of neural tube defects in Northern and Eastern Europe and of orofacial clefts in Scandinavian populations (with Finland a very significant outlier for cleft palate) may be partly responsible, as also may inclusion of a higher proportion of less severe congenital heart defects in some registers. Most of the remaining differences appear to be due to differences in ascertainment. Under-ascertainment is considered likely if a registry reports less than 20/1000 total congenital anomalies (EUROCAT 2011), which corresponds to around 17/1000 “non-genetic” anomalies. Using this criterion, under-ascertainment may be suspected in Croatia, Italy, Spain and Portugal (Table 1).

- Step 3: Calculating baseline rates by system group

The pregnancy outcomes may vary depending on the type of malformation (i.e. system group affected) and if it was isolated or associated with malformations in other systems. Consequently, baseline prevalence by system group is valuable in assessing outcomes. EUROCAT data available publically on the Internet (EUROCAT) only relate to non-syndromic congenital malformations by system group, meaning that individuals with more than one malformation are represented multiple times. A data request could have been made directly to EUROCAT for this data. However, in order to use the publically available data to obtain rates for affected individuals by system group, we needed to estimate what proportion of individuals had an isolated malformation (not associated with a malformation in another system), and to add a “multiple malformation” group. This was done in three steps.

- Estimate proportion of isolated and multiple malformations

The proportion of NSCM that was isolated and the proportion that contributed to multiple malformations were estimated by comparing births of affected individuals with the total sum of the number of malformations across all the systems groups (Table 2). The difference between these totals is an indicator of the frequency of multiple malformations. Overall, 19.56 [95% CI 19.47, 19.56] per 1000 births are affected by non-syndromic malformations, whereas the sum of malformations by system group is 20.21 [95% CI 20.12, 20.3] per 1000 births. The difference between the two (2.8/1000) represents the frequency of multiple malformations.

- Estimate the baseline prevalence rate for multiple malformation group

**Table 2:** Non-syndromic malformations by system group, birth prevalences and birth outcomes 2000–2009 inclusive (data downloaded Jan 2015)

Malformation group	Rates /1000 [95% CI]				Foetal death % of pregnancies intended to continue
	Live births	Fetal deaths	TOPFA	Total affected births	
Neural tube defects (NTD)	0.26 [0.25,0.27]	0.04 [0.04,0.04]	0.65 [0.63,0.67]	0.95 [0.93,0.97]	13.33
Central nervous system not NTD	0.86 [0.84,0.88]	0.04 [0.04,0.04]	0.35 [0.34,0.36]	1.25 [1.23,1.27]	4.44
Eye	0.38 [0.37,0.39]	0.01 [0.01,0.01]	0.04 [0.04,0.04]	0.43 [0.42,0.44]	2.56
Ear, face and neck	0.31 [0.3,0.32]	0.01 [0.01,0.01]	0.05 [0.05,0.05]	0.37 [0.36,0.38]	3.13
Congenital heart defects	6.59 [6.54,6.64]	0.07 [0.06,0.08]	0.37 [0.36,0.38]	7.03 [6.98,7.08]	1.05
Respiratory	0.26 [0.25,0.27]	0.03 [0.03,0.03]	0.11 [0.1,0.12]	0.4 [0.39,0.41]	10.34
Oro-facial clefts	1.25 [1.23,1.27]	0.02 [0.02,0.02]	0.11 [0.1,0.12]	1.38 [1.36,1.4]	1.57
Digestive system	1.42 [1.4,1.44]	0.04 [0.04,0.04]	0.17 [0.16,0.18]	1.63 [1.6,1.66]	2.74
Abdominal wall defects	0.33 [0.32,0.34]	0.02 [0.02,0.02]	0.16 [0.15,0.17]	0.51 [0.5,0.52]	5.71
Urinary	2.78 [2.75,2.81]	0.05 [0.05,0.05]	0.37 [0.36,0.38]	3.2 [3.16,3.24]	1.77
Genital	1.99 [1.96,2.02]	0.01 [0.01,0.01]	0.07 [0.06,0.08]	2.07 [2.04,2.1]	0.50
Limb	3.78 [3.74,3.82]	0.06 [0.06,0.07]	0.29 [0.28,0.3]	4.13 [4.09,4.17]	1.56
Total sum of malformations	20.21 [20.12,20.3]	0.4 [0.39,0.41]	2.96 [2.93,2.99]	23.57 [23.47,23.67]	1.94
Rate for individuals with non-syndromic malformations	17.41 [17.33,17.49]	0.26 [0.25,0.27]	1.92 [1.90,1.95]	19.56 [19.47,19.65]	1.45
Difference between total sum and rate for individuals with NSCM	2.8 [2.77,2.83]	0.14 [0.13,0.15]	1.04 [1.02,1.06]	3.98 [3.94,4.02]	
Individuals with isolated malformations, % of system sum	86.15 [86.12,86.17]	65.01 [64.51,65.5]	64.87 [64.68,65.05]	83.11 [83.08,83.15]	

*TOPFA*: termination of pregnancy for foetal anomaly,

*NSCM*: non-syndromic congenital malformation,

*NTD*: neural tube defects,

*CI*: confidence interval

To derive a baseline prevalence rate for the multiple malformation group, we used an average of estimated rates reported in the literature. Average reported rates include: 1.27 /1000 reported by Rittler et al. (2008), 1.59/1000 reported by Garne et al. (2011) and 1.58 reported by Calzolari et al. (2014). Slightly lower rates of 1.08/1000 for multiple malformations, with 12.7% foetal deaths and 27% terminations of pregnancy were reported by Tennant et al. (2010): however, this report excludes most genital and limb malformations and covered a period (1985–2003) when prenatal diagnosis was evolving. In MGDb, we use 1.41/1000 multiple malformations as it is an average of the reported rates in the literature (Garne et al. 2011; Rittler et al. 2008).

- Calculating rates for isolated malformations by system group

In order to obtain rates for isolated malformations by system group, the proportion within each malformation group contributing to multiple malformations needed to be assessed and rates adjusted accordingly. EUROCAT non-syndromic total births/1000 were adjusted using the percentage reported in the literature to be associated with other malformations. Published association rates from Garne et al. (2011) supplemented by rates from Rittler et al. (2008) were used to estimate within each system group, those which are isolated and those which contribute to multiple malformations. Table 3 shows the percentage associated with other malformations for each system group and the adjusted baseline birth prevalence rates by malformation group.

**Table 3:** Adjusted baseline rates for different malformation groups. Baseline rates were adjusted based on association rates obtained from Garne et al. 2011, supplemented with data from Rittler et al. 2008 (shown in italics)

Group	EUROCAT non-syndromic total births/1000	Per cent associated with other malformations	Total births, isolated malformations/1000	Total births, non-isolated malformations/1000
Non-syndromic malformations	19.56 [19.47,19.65]		18.40	1.41
Neural Tube Defects (NTD)	0.95 [0.93,0.97]	13.00	0.83	0.12
Central nervous system not NTD	1.25 [1.23,1.27]	*23.50*	0.96	0.29
Eye	0.43 [0.42,0.44]	*24.40*	0.33	0.10
Ear, face and neck	0.37 [0.36,0.38]	*38.10*	0.23	0.14
Congenital heart defects^a^	7.03 [6.98,7.08]	11.00	6.26	0.77
Respiratory	0.4 [0.39,0.41]	*11.70*	0.35	0.05
Oro-facial clefts^a^	1.38 [1.36,1.4]	15.50	1.17	0.21
Digestive system	1.63 [1.6,1.66]	*29.90*	1.14	0.49
Abdominal wall defects	0.51 [0.5,0.52]	14.20	0.44	0.07
Urinary	3.2 [3.16,3.24]	*10.70*	2.86	0.34
Genital	2.07 [2.04,2.1]	12.80	1.81	0.26
Limb	4.13 [4.09,4.17]	*11.00*	3.68	0.45

^a^Country-specific rates were used NTDs and orofacial clefts in MGDb, rates were obtained from registries or the published literature. European average rates were calculated for congenital heart defects using data from a EUROCAT special report on CHD

## Estimating birth outcome rates for isolated malformations

Data are available in the EUROCAT database for outcomes of affected pregnancies for all non-syndromic malformations combined. We adjusted outcome rates for isolated and multiple malformations in the same way as for calculating birth prevalences, assuming that isolated malformations accounted for 86.15% of live births, 65.01% of terminations and 64.87% of foetal deaths (Table 2). Table 4 shows the resulting estimates for isolated malformations by system group. We have done this in the absence of more refined data. As pregnancy outcomes may vary depending on the type of defect (i.e. system group affected), this may result in under/over estimation of outcomes for certain system sub-groups.

**Table 4:** Adjusted rates for outcomes of different malformation groups**.** The approximate distribution of birth outcomes for isolated congenital malformation groups was calculated from rates for non-syndromic malformation groups assuming that isolated malformations account for 86.15% of live births, 65.01% of terminations and 64.87% of foetal deaths

Malformation group	Non-syndromic/1000				Isolated/1000				
	Total	Live births	Foetal deaths	TOPFA	Total	Live births	Foetal deaths	TOPFA	Foetal death % of pregnancies intended to continue
Births with non-syndromic malformations	19.8	17.9	0.25	1.66					1.45
Births with multiple malformations	1.41	0.97	0.07	0.37					7.0
Neural tube defects (NTD)	0.95	0.26	0.04	0.65	0.83	0.22	0.03	0.42	10.37
Central nervous system not NTD	1.25	0.86	0.04	0.35	0.97	0.74	0.03	0.23	3.38
Eye	0.43	0.38	0.01	0.04	0.33	0.33	0.01	0.03	1.94
Ear, face and neck	0.37	0.31	0.01	0.05	0.23	0.27	0.01	0.03	2.37
Congenital heart defects	7.03	6.59	0.07	0.37	6.26	5.69	0.05	0.24	0.79
Respiratory	0.4	0.26	0.03	0.11	0.35	0.22	0.02	0.07	7.99
Oro-facial clefts	1.38	1.25	0.02	0.11	1.17	1.08	0.01	0.07	1.19
Digestive system	1.63	1.42	0.04	0.17	1.14	1.23	0.03	0.11	2.08
Abdominal wall defects	0.51	0.33	0.02	0.16	0.44	0.29	0.01	0.10	4.36
Urinary	3.2	2.78	0.05	0.37	2.86	2.40	0.03	0.24	1.34
Genital	2.07	1.99	0.01	0.07	1.81	1.72	0.01	0.05	0.38
Limb	4.13	0.29	0.06	3.78	3.68	0.19	0.04	3.27	1.18

*TOPFA* termination of pregnancy for foetal anomaly

Foetal deaths are difficult to estimate due to possible under-ascertainment and their relationship to TOPFA. We have assumed that the foetal death rates calculated as a proportion of continuing pregnancies are unlikely to be greatly affected by differences in the level of pregnancy care. However, this method may lead to under-estimation of foetal death in the absence of care, because the most severe cases are both most likely to be detected on prenatal ultrasound scan with many families opting for termination of pregnancy based on predicted poor outcomes, and more likely to result in foetal death in the absence of termination of pregnancy. EUROCAT country rates for total foetal deaths for non-genetic conditions are shown in Table 5. Countries are ranked in descending order of foetal deaths/1000 births. As can be seen, there are wide inter-country differences. The high rate reported from Ukraine reflects persisting high prevalence of neural tube defects, but this does not apply for other countries. If inter-country differences were due to selective termination of pregnancies otherwise most likely to end in foetal death, it would be expected that the foetal death rate would fall as rate for termination of pregnancy rises. However, the very weak relationship between the two (web appendix Fig. 2) suggests under-ascertainment of termination of pregnancy and/or foetal deaths in some registries as the likely explanation for most of the inter-country differences.

**Table 5:** Non-genetic congenital malformations. Foetal death rates by country, ranked in descending order of % of continuing pregnancies. Data source: EUROCAT 2000–2009

Country	Foetal deaths/1000	Foetal deaths % of total	Foetal deaths % of continuing pregnancies
Ukraine	0.80	3.64	4.19
Ireland	0.58	3.43	3.43
Malta	0.57	2.14	2.14
United Kingdom	0.48	2.44	2.81
Denmark	0.43	1.77	1.93
Germany	0.43	1.37	1.46
Finland (assoc)	0.43	1.14	1.21
Netherlands	0.35	1.67	1.74
Belgium	0.31	1.51	1.64
Switzerland	0.30	0.97	1.06
Austria	0.29	0.98	1.03
France	0.26	1.28	1.51
Norway	0.21	0.68	0.73
Croatia	0.17	1.07	1.13
Portugal	0.15	1.66	1.79
Spain	0.11	0.97	1.04
Czech Rep (assoc)	0.11	0.34	0.39
Poland	0.10	0.66	0.66
Hungary	0.09	0.35	0.37
Italy	0.07	0.44	0.50
Sweden (assoc)	0.05	0.29	0.32
Average for full registries TOP legal reported	0.26	1.30	1.40

*assoc: associated registry

## Strengths and limitation of European reference rates

Using publically available EUROCAT data to obtain reference rates has a number of strengths including representation of a wide range of countries across Europe, participating countries having advanced facilities for diagnosis and data collection, collection of data on a wide range of disorders in a standardised manner and reporting of all pregnancy outcomes including foetal death and elective termination of pregnancy for foetal anomaly.

From a practical view, data accessibility and the distinction of congenital malformations by cause, further enable ease of use for this purpose. However, there are limitations, with individual registries differing in case ascertainment due to a number of factors (e.g. resources, extent, amount of prenatal diagnosis available to women, number of data sources used and mode of access to records etc.). This is particularly likely for outcomes that end in a foetal loss or termination of pregnancy and this may partially account for considerable differences between countries in reported rates. Nevertheless, the large number of participating registries tends to reduce inter-country biases when calculating European averages. Many of these limitations could have been overcome by requesting data directly from EUROCAT and using the EUROCAT data quality indicators (Loane et al. 2011) to only select those registers that have good ascertainment and high-quality data.

The differential contribution of country data to EUROCAT can affect calculated European averages as countries with particularly high or low recorded rates could significantly skew the average, e.g. the inclusion of registries that do not report terminations of pregnancy would falsely reduce average rates whereas the inclusion of countries where termination of pregnancy is illegal or not reported would reduce estimated average rates for termination of pregnancy. We have attempted to control for the impact of TOPFA, by excluding countries that do not report on this subject and exclude countries where TOPFA is illegal when looking at pregnancy outcomes.

We also tested the potential effect of differences in population size on the country rates by adjusting the rates in Table 2 using World Population Prospects (WPP) annual births data. The result was an increase of approximately 5% in average baseline birth prevalence, a 9% increase in terminations and a 6% increase in foetal deaths (see web appendix Table 2). In principle, these rates could be used to adjust average EUROCAT rates. However, no adjustment is currently made, in order to minimise complication in the methodology and to remain as close as possible to the primary data. Furthermore, this may lead to over-estimation of the results which we wished to avoid.

It is recognised that a large proportion of congenital malformations have an unknown cause (Feldkamp et al. 2017). The diversity and relatively constant birth prevalence of these unexplained malformations suggest that many are due to random accidents during the complex process of embryonic development, a concept that is supported by mathematical considerations (Kurnit et al. 1987). There have been few global studies carrying out detailed comparisons of rates in populations with different ethnicities. Studies have been carried out in the United States and Europe report ethnic differences in neural tube defects, orofacial clefts, thyroid a/dysplasia and polydactyly (Bundey and Alam 1993; Chitty and Winter 1989; Egbe 2015). In addition, EUROCAT rates for France include data from congenital anomaly registries in Martinique, Guadeloupe and Reunion, all with predominantly non-European populations, reported rates are similar to those for metropolitan France. Due to limited evidence to indicate significant country differences in birth prevalence of these disorders (apart from neural tube defects and orofacial clefts), robust data from high-income countries could be applied to those where there is a lack of data to obtain provisional estimates.

Apart from folic acid supplementation or food fortification (which is not yet policy in most of Europe), the main intervention likely to cause true inter-country variation in the observed birth prevalence of congenital malformations is the level of access to prenatal diagnosis with the option of termination of pregnancy. The birth prevalence rates developed here can be used as provisional estimates for the overall baseline prevalence of certain congenital malformations and for estimating the proportions of each malformation group that end in termination of pregnancy or foetal death.

Other sources of data including the International Clearing house for Birth Defect Surveillance and Research (ICBDSR) and National Birth Defects Prevention Network (NBDPN) were also assessed for potential inclusion. An advantage of ICBDSR is that it includes a spectrum from non-European countries. However, ICBDSR includes data from a diverse range of data systems and there is less consistency in data collection across programmes that contribute to ICBDSR, this can lead to under-ascertainment (see Table 3 web appendix). Detailed data from NBDPN is not available online; furthermore, analysis of available data suggests that the reported rates are higher than EUROCAT (Web appendix Table 4). In the interests of providing a conservative estimate, we have employed data from EUROCAT. In addition, the relative standardisation of EUROCAT data was an advantage.

EUROCAT averages provide a useful baseline for policy and planning purposes for most non-syndromic malformations in countries where high-quality registry data are not yet available. However, ethnic and environmental factors affect the birth prevalence of some groups of non-syndromic malformations, including neural tube defects and orofacial clefts. For these conditions, local or regional information on prevalence where available is preferable to EUROCAT averages, and EUROCAT and ICBDSR country-specific data has been used in MGDb (Kadir et al. 2016). Our aim was to enable non-specialist policy makers to develop estimates; hence, we have developed methods that rely on readily accessible data sources. These estimates can be improved on and can be done so through direct contact with data sources such as EUROCAT to obtain more precise breakdowns.

## Conclusion

Accurately assessing the prevalence of pregnancies and births affected by congenital malformations requires clinical expertise, advanced diagnostic techniques including routine foetal anomaly scanning and availability of perinatal/paediatric post-mortem examination, coupled with medically certified registration of cause of death and robust reporting to dedicated population-based congenital anomaly registers. These resources are not available in the majority of countries outside Europe and North America. In these countries, diagnosis frequently relies primarily on physical examination, with only around 30% of congenital malformations can be reliably diagnosed (Neel 1958; Todros et al. 2001). In addition, where available registries are usually hospital-based and cover the first few days of life only, thus limiting prevalence rate estimates.

Based on our analysis of EUROCAT registry data, there is limited evidence to indicate significant country differences in the baseline birth prevalence of non-syndromic congenital malformations, other than neural tube defects and orofacial clefts. We therefore propose the use of methods based on rates observed in high-income European settings, to obtain provisional global, regional and country estimates for the birth prevalence of these malformation groups in settings with no data, as a starting point. These methods do not provide precise estimates, but those that may be sufficient for policy and programmatic purposes, until robust data become available for all settings; which is only possible through dedicated and sustainable surveillance systems. Many WHO regions including South East Asia, Africa and Latin America have recognised this issue and are working to develop capacity in this area as part of their framework for the prevention and control of birth defects (Flores et al. 2015; WHO Regional Office for South East Asia; WHO Regional Office for South East Asia 2013).

## Electronic supplementary material

ESM 1 (DOCX 28 kb)

## Abbreviations

CHD: Congenital Heart Disease
EUROCAT: European Surveillance of Congenital Anomalies and Twins
ICBDSR: International Clearinghouse for Birth Defects Surveillance and Research
MGDb: Modell Database of Congenital disorders
NSCM: Non-syndromic congenital malformations
OFC: Orofacial cleft
TOPFA: Termination of pregnancy for fetal anomaly
UN WPP: United Nations World Population Prospects
