# Supplementary material for: Estimating the birth prevalence and pregnancy outcomes of congenital malformations worldwide
Source: J Community Genet. 2018 Sep 14;9(4):387–96. doi: 10.1007/s12687-018-0384-2 (PMC6167261; doi:10.1007/s12687-018-0384-2)
Supplement: Supplementary file 1 — (DOCX 28 kb) [file 12687_2018_384_MOESM1_ESM.docx]

**Online Resources**

Table 1 “Other” diagnostic groups, rates /1,000 births (EUROCAT average data for 2000-09)

| Diagnostic group |  |  | Outcome, % of group | | | Outcomes /1,000 births | | |
| --- | --- | --- | --- | --- | --- | --- | --- | --- |
|  | **Births /1,000** | **% of total "other"** | **Live birth** | **Fetal death** | **TOPFA** | **Live birth** | **Fetal death** | **TOPFA** |
| **Teratogenic syndromes with malformations** | 0.111 | 6.8 | 81 | 4 | 15.6 | 0.09 | 0.004 | 0.017 |
| **Maternal infections resulting in malformations** | 0.051 | 3.1 | 67.3 | 5.4 | 27.6 | 0.034 | 0.003 | 0.014 |
| **Fetal alcohol syndrome** | 0.037 | 2.3 | 97.8 | 0.8 | 1.6 | 0.036 | 0 | 0.001 |
| **Valproate syndrome** | 0.009 | 0.5 | 89.5 | 0 | 9.3 | 0.008 | 0 | 0.001 |
| **Total Environmental disorders** | 0.208 | 12.7 | 81 | 3.6 | 15.8 | 0.168 | 0.007 | 0.033 |
| **Genetic syndromes & micro-deletions** | 0.472 | 28.9 | 83.5 | 2.6 | 14.2 | 0.394 | 0.012 | 0.067 |
| **Congenital skin disorders** | 0.291 | 17.8 | 96.2 | 0.8 | 3.5 | 0.28 | 0.002 | 0.01 |
| **Sequences** | 0.202 | 12.3 | 61.2 | 6.3 | 32.8 | 0.124 | 0.013 | 0.066 |
| **Skeletal dysplasias** | 0.173 | 10.6 | 51.7 | 3.8 | 44.6 | 0.089 | 0.007 | 0.077 |
| **Craniosynostosis** | 0.165 | 10.1 | 97 | 0.5 | 2.8 | 0.16 | 0.001 | 0.005 |
| **Situs inversus** | 0.062 | 3.8 | 79 | 0.8 | 20.3 | 0.049 | 0 | 0.013 |
| **Congenital constriction bands /amniotic band** | 0.044 | 2.7 | 47.8 | 13.8 | 38.8 | 0.021 | 0.006 | 0.017 |
| **Conjoined twins** | 0.019 | 1.1 | 17.3 | 8.1 | 73.5 | 0.003 | 0.002 | 0.014 |
| **Total other not environmental** | 1.427 | 87.3 | 78.5 | 3 | 18.8 | 1.12 | 0.043 | 0.268 |
| **Total other diagnoses /1,000** | 1.635 | 100 | 78.8 | 3.1 | 18.4 | 1.288 | 0.051 | 0.301 |

Figure 1 Evolution of rates for individuals with total, non-genetic and genetic congenital anomalies /1,000 births by 5-year intervals. Average for all full registries minus Poland. The rise in genetic cases is largely due to increasing proportion of older mothers. Rates for 2000-09 are shown separately, because this is the interval on which EUROCAT average rates are calculated.

Figure 2 Relation of reported rates for total terminations of pregnancy and total fetal deaths. (Ukraine, Malta, Ireland, Poland excluded.)

Table 2. Effect on European average rates for non-genetic anomalies, of adjusting for population size (number of births)

|  |  | Outcomes/1,000 | | | Outcomes % | | |
| --- | --- | --- | --- | --- | --- | --- | --- |
| **Non-syndromic "all anomalies"** | **Total /1,000** | **TOPFA /1,000** | **Fetal deaths /1,000** | **Live births /1,000** | **% TOPFA** | **% Fetal deaths** | **% Live births** |
| **EUROCAT data** | 20.0 | 1.89 | 0.34 | 17.8 | 9.45 | 1.70 | 88.8 |
| **Adjusted for pop size** | 21.1 | 2.07 | 0.36 | 18.6 | 9.82 | 1.72 | 88.5 |
| **Increase with adjustment** | 1.06 | 0.18 | 0.02 | 0.86 | 0.37 | 0.01 | -0.38 |
| **% increase with adjustment** | 5.3 | 9.4 | 6.1 | 4.8 | 3.89 | 0.76 | -0.43 |

# Table 3 Comparison of congenital malformation groups reported by EUROCAT and ICBDSR

| Malformation group | EUROCAT data | | | | ICBDSR data | |
| --- | --- | --- | --- | --- | --- | --- |
|  | Births /1,000 non-chromosomal | % of total non-chr | Named diagnoses /1,000 | Named diagnoses % of malfn group | ICBDMR births /1,000 est | % of EUROCAT diagnoses in ICBDMR |
| Nervous system | 1.82 | 9 | 1.48 | 81 | 1.48 | 81 |
| Eye | 0.32 | 2 | 0.20 | 62 | 0.10 | 32 |
| Ear, face and neck | 0.43 | 2 | 0.03 | 7 | 0.03 | 7 |
| Congenital heart disease | 5.73 | 29 | 6.23 | 109 | 0.90 | 16 |
| Respiratory | 0.44 | 2 | 0.11 | 24 | 0.07 | 15 |
| Oro-facial clefts | 1.35 | 7 | 1.35 | 100 | 1.35 | 100 |
| Digestive system | 1.27 | 6 | 0.92 | 73 | 0.81 | 64 |
| Abdominal wall defects | 0.40 | 2 | 0.38 | 95 | 0.38 | 95 |
| Urinary | 2.41 | 12 | 1.31 | 54 | 0.00 | 0 |
| Genital | 1.57 | 8 | 1.27 | 81 | 1.27 | 81 |
| Limb | 3.46 | 17 | 3.33 | 96 | 0.57 | 16 |
| Other malformations | 0.79 | 4 | 0.53 | 67 | 0.00 | 0 |
| Total (sum) | 19.98 | 100 | 17.13 | 86 | 6.96 | 35 |

Table 4 Comparison of reported rates by malformation groups for period 2000-5. Rates for all North American registries are higher than the EUROCAT average, consistent with the conclusion that this is a low estimate.

| EUROCAT GROUPS | EUROCAT av. for recorded anomalies | North America | | | | | |
| --- | --- | --- | --- | --- | --- | --- | --- |
|  |  | Canada Alberta | Canada National | Canada British Columbia | USA Texas | USA Utah | USA Atlanta |
| CNS | 1.48 | 1.86 | 1.53 | 2.66 | 2.18 | 1.51 | 2.05 |
| OFCs | 1.35 | 2.02 | 1.67 | 1.76 | 1.60 | 2.13 | 1.44 |
| Eye | 0.10 | 0.17 | 0.10 | 0.11 | 0.28 | 0.17 | 0.27 |
| Ear, face,neck | 0.03 | 0.06 | 0.00 | 0.07 | 0.03 | 0.01 | 0.03 |
| CHD | 0.99 | 1.31 | 1.73 | 1.52 | 1.46 | 1.93 | 1.73 |
| Respiratory | 0.07 | 0.11 | 0.26 | 0.20 | 0.11 | 0.02 | 0.04 |
| Digestive | 0.81 | 1.42 | 1.46 | 1.53 | 1.10 | 1.05 | 1.01 |
| Genital | 1.27 | 2.24 | 2.97 | 2.05 | 1.75 | 0.42 | 0.91 |
| Urinary | 0.21 | 0.68 | 0.64 | 0.20 | 0.25 | 0.39 | 0.15 |
| Limb | 1.36 | 2.54 | 1.78 | 1.46 | 0.85 | 0.64 | 0.71 |
| Abd wall | 0.38 | 0.68 | 0.56 | 0.82 | 0.68 | 0.75 | 0.51 |
| Sum recorded groups | 8.1 | 13.1 | 12.7 | 12.4 | 10.3 | 9.0 | 8.8 |
| % of EUROCAT sum |  | 162.5 | 157.8 | 153.7 | 127.7 | 111.8 | 109.7 |
|  |  |  |  |  |  |  |  |
| Sum minus NTDs & OFCs | 5.2 | 9.2 | 9.5 | 8.0 | 6.5 | 5.4 | 5.4 |
| % of EUROCAT sum | 100 | 176.4 | 182.3 | 152.5 | 124.5 | 102.9 | 102.5 |
|  |  |  |  |  |  |  |  |
| Births 1,000s |  | 235.1 | 2,021.8 | 244.1 | 1,870.4 | 297.9 | 308.0 |
